# Physicochemical Attributes of Solid Hyaluronic Acid Technology Platform-Based Film Drive Rapid Oral Permeation and Barrier Enhancement via LKB1/CaMKKβ-AMPK Signaling

**DOI:** 10.1021/acsomega.6c00470

**Published:** 2026-05-18

**Authors:** Ha-Young Park, Soo-Bin Shin, Dong-Keon Kweon

**Affiliations:** † Advanced Radiation Technology Institute, 65405 Korea Atomic Energy Research Institute, Jeongeup 56212, Republic of Korea; ‡ Department of Integrative Food, Bioscience and Biotechnology, Chonnam National University, Gwangju 61186, Republic of Korea; § Jinwoo Bio Co., Ltd., Giheung-gu, Yongin 17111, Republic of Korea

## Abstract

Hyaluronic acid (HA) is widely used in oral film systems; however, the functional interactions of excipient-free HA films with oral epithelial tissues, particularly with respect to permeability and barrier modulation, remain insufficiently understood. In this study, we investigated the physical properties, permeation characteristics, and oral epithelial barrier responses of a solid hyaluronic acid technology platform (SHTP) film composed exclusively of HA, using buccal epithelial TR146 cell layers and a rat model. Compared with a commercial HA film containing excipients, the SHTP film exhibited distinct hydration-driven properties, including rapid water uptake (815% within 60 s) and enhanced swelling capacity. These properties primarily facilitated rapid buccal HA permeation, as reflected by increased transepithelial flux and higher apparent permeability coefficients (P_app_). Following this initial permeation-enhancing effect, the SHTP film reinforced epithelial barrier integrity, as evidenced by reduced paracellular transport of fluorescein isothiocyanate-dextran (FD4) and increased transepithelial electrical resistance (TEER). Mechanistic analyses suggested that this barrier-supportive response was associated with the upregulation of tight junction-related gene expression and activation of the LKB1/CaMKKβ-AMPK signaling pathway. *In vivo* studies further demonstrated rapid oral buccal absorption and subsequent systemic distribution of HA delivered via the SHTP film. Collectively, these findings demonstrate that excipient-free HA films exert a sequential dual effect, facilitating rapid transepithelial permeation, followed by reinforcement of epithelial barrier integrity. This work provides mechanistic insight into hydration-driven interactions between HA and the oral epithelium and supports the application of excipient-free HA films in oral biomedical and epithelial barrier-oriented delivery systems.

## Graphical abstract

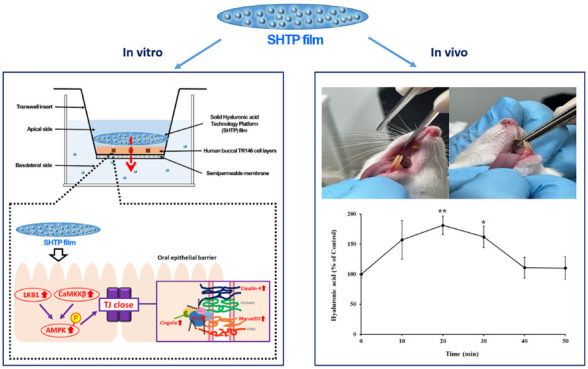

## 1 Introduction

Hyaluronic acid (hyaluronan, HA) is a well-known linear and unbranched biopolymer composed of repeating units of d-glucuronic acid and N-acetyl-d-glucosamine linked by alternating β1–4 and β1–3 glycosidic bonds.^1,2^ Traditionally applied in biomedical fields, HA has recently attracted increasing interest in the functional food and nutraceutical sectors owing to its excellent biocompatibility, hydration-retaining capacity, and barrier-supportive properties. It plays crucial roles in maintaining tissue hydration, wound healing, and mucosal regeneration.^3^ Based on these biofunctional characteristics and its capacity to interact with mucosal surfaces, HA is being explored as a food-grade biopolymer for developing functional matrices and mucosal delivery platforms targeting both local oral and systemic health benefits.^4–6^

Oral mucosal delivery offers distinct advantages over conventional administration routes, including bypassing first-pass metabolism, rapid absorption, and improved patient compliance. The buccal and sublingual mucosa are particularly attractive, providing both paracellular (via intercellular spaces) and transcellular (via apical and basolateral membranes) transport pathways for bioactive compounds. Notably, buccal permeability has been reported to be 4–4000 times higher than that of the skin, underscoring its potential as an efficient absorption route. Accordingly, the oral cavity has been utilized as a site for local and systemic delivery of nutraceutical and functional ingredient using various dosage forms, including adhesive gels, films, and patches.^4,7,8^ Nevertheless, effective transport across the oral epithelium remains limited by epithelial barrier properties that are not yet fully understood.

Oral films represent versatile polymeric matrices that can serve as efficient platforms for the controlled release and mucosal delivery of bioactive compounds. HA-based oral films have been extensively investigated due to their mucoadhesive behavior and favorable biocompatible.^9,10^ Recent formulations have incorporated excipients such as pullulan, glycerin, and modified starch to enhance mechanical flexibility and dissolution characteristics.^10–14^ However, the inclusion of such excipients may also interfere with bioactive release kinetics or epithelial transport by forming complex polymer networks that delay hydration or alter mucosal interactions.^15–17^

Recent advances in polymer engineering have demonstrated that the physical properties of oral films, including tensile strength, elongation at break, mucoadhesive strength, and water absorption, critically influence hydration behavior, mucosal interaction, and subsequent permeation efficiency. In this context, the solid hyaluronic acid technology platform (SHTP) film, composed exclusively of HA without any excipients, exhibited distinct physicochemical properties compared with commercial HA films, including higher tensile strength and greater elongation at break, markedly faster hydration, and relatively lower mucoadhesive strength. These characteristics suggest that the absence of excipients yields a rapidly hydrating yet mechanically robust matrix that may promote faster HA release and epithelial transport. However, whether these physicochemical differences translate into enhanced buccal permeability or distinct epithelial barrier-modulating responses has not been systematically investigated.

In this study, we investigated the permeation behavior and underlying mechanisms of oral buccal absorption of an HA polymer film composed entirely of highly purified HA without excipients, referred to as the solid hyaluronic acid technology platform (SHTP) film. We evaluated its permeation efficiency across buccal epithelial layers and compared it with that of commercially available HA films containing excipients such as pullulan, glycerin, and modified starch. In parallel, we assessed its impact on oral epithelial barrier function by examining changes in tight junction (TJ)-related gene expression and barrier integrity. Furthermore, we analyzed the involvement of AMP-activated protein kinase (AMPK) signaling and its upstream regulators, liver kinase B1 (LKB1) and calcium/calmodulin-dependent protein kinase kinase β (CaMKKβ), in TJ regulation. Finally, *in vivo* buccal absorption studies in rats were conducted to validate the translational potential of the SHTP platform as an excipient-free oral delivery and epithelial barrier-oriented therapeutic system.

## 2 Materials and Methods

### 2.1 Materials

A SHTP-based film (SHTP film) was obtained from Jinwoo Bio Co., Ltd. (Yongin, Republic of Korea).^18,19^ The SHTP film contains 14 mg/cm^2^ of HA and is composed exclusively of HA without the inclusion of additional excipients. The molecular weight of HA used for SHTP film preparation falls within the low-molecular-weight range (<10 kDa), as confirmed by gel permeation chromatography (GPC) analysis of the starting material, which showed a peak molecular weight (M_p_) of approximately 5.4 kDa (5392 Da) (Figure S1). The SHTP film was manufactured using a continuous casting and controlled drying process to form a self-supporting HA matrix, as described in a previous patent.^18^ The drying conditions were controlled to preserve structural integrity and mechanical robustness without the need for chemical plasticizers. For comparison, a commercial HA film containing 32 mg/cm^2^ of HA and excipients (pullulan, glycerin, and modified starch) was tested. The molecular weight of HA in the commercial film also falls within the low-molecular-weight range (<29 kDa), based on the manufacturer’s specification. Fluorescein isothiocyanate-dextran 4000 Da (FD4) was purchased from Sigma-Aldrich (St. Louis, MO, USA). All other reagents were of analytical grade and were used without further purification.

### 2.2 Physical Properties of Films (Tensile Strength, Elongation at Break, Mucoadhesive Strength, and Water Absorption)

Tensile strength (MPa) and elongation break (%) of the films were determined using a universal testing machine (Instron, Norwood, MA, USA) equipped with a 500 N load cell, following the ASTM D822–18 standard.^20^ Films were mounted with an initial grip separation of 50 mm and stretched at a crosshead speed of 50 mm/min until failure. All tests were performed under controlled environmental conditions (23 ± 2 °C, 45 ± 5% relative humidity). Tensile strength was calculated by dividing the maximum force at break by the cross-section area of the film, and elongation at break was calculated at the ratio of extension at break to the initial length. Mucoadhesive strength of the HA films were evaluated using a universal testing machine (QMESYS, Gyeonggi-do, Republic of Korea) equipped with a 10 kgf load selector. Film were fixed to a flat prove and brought into contact with a mucosal substrate for a defined contact time. The probe was then withdrawn at a crosshead speed of 50 mm/min, and the maximum detachment force (peak force required to separate the film form the substrate), was recorded. Water absorption of HA films was assessed by an immersion method. Film samples were immersed in distilled water for 1 min and weighed immediately after removal. Water absorption (%) was calculated based on the increase in sample weight using eq 1:

1 $$\mathrm{Water}\;\mathrm{absorption}\;\left(\%\right)=\frac{W-W_{\mathrm{initial}}}{W_{\mathrm{initial}}}\times 100$$

where W and W_initial_ represent the weights of the samples after immersion and before immersion, respectively.

### 2.3 Culture and Maintenance of TR146 Cells

The human buccal carcinoma cell line TR146 (ECACC #10032305) was obtained from Public Health (London, UK). Cells were cultured in Ham’s F-12 (Sigma-Aldrich) supplemented with 10% fetal bovine serum (FBS; Hyclone, Logan, UT, USA), 2 mM l-glutamine, 100 U/mL penicillin, and 100 μg/mL streptomycin (GIBCO, Gaithersburg, MD, USA). Cultures were maintained at 37 °C in a humidified incubator with 5% CO_2_, and the medium was refreshed every other day. Cells were seeded at a density of 1–2 × 10^4^ cells/cm^2^ in appropriate culture vessels. When reaching 70–80% confluence, cells were detached using 0.25% trypsin/EDTA (GIBCO). For all experiments, only TR146 cells at passages 13 to 19 were utilized.

### 2.4 HA Permeability Assay

TR146 cells were seeded at a density of 5 × 10^4^ cells/well in 12-well transwell inserts (Corning Inc.) with a polyethylene terephthalate membrane (0.9 cm^2^ growth area and 0.4 μm pore diameter) for the HA permeability assay. The cells were allowed to grow and differentiate to form confluent cell layers for 21–28 days and the culture medium was replaced 2–3 times per week. After formation of the cell layer, the cells were washed twice with Hank’s balanced salt solution (HBSS, pH 7.4, GIBCO) and incubated for 10 min for equilibration. HA film samples (SHTP film and commercial HA film) with an area of 0.87 cm^2^ were placed on the apical side of the transwell, followed by the addition of HBSS (0.5 mL) to initiate film dissolution. At time points of 0.25, 0.5, 1, 2, 4, 8, and 24 h, 120 μL was collected from the basolateral side (1.5 mL) and immediately replaced with an equal volume of HBSS (pH 7.4) to maintain constant volume. The amount of HA that permeated through the TR146 cell layers was quantified using a hyaluronan enzyme-linked immunosorbent assay kit (HA-ELISA, Echelon Biosciences, Inc., Salt Lake City, UT, USA). The permeation and transepithelial flux of HA were calculated based on eqs 2 and 3, respectively:

2 $$\mathrm{Permeation amount}\;\left(\mathrm{ng}/{\mathrm{cm}}^{2}\right)=\mathrm{transported HA amount}\;\left(\mathrm{ng}/\mathrm{mL}\right)\times \mathrm{receiver volume}\;\left(\mathrm{mL}\right)/\mathrm{surface area}\;\left({\mathrm{cm}}^{2}\right)$$

3 $$\mathrm{Transepithelial flux of HA}\;\left(\mathrm{ng}/{\mathrm{cm}}^{2}/h\right)=\mathrm{permeation amount}\;\left(\mathrm{ng}/{\mathrm{cm}}^{2}\right)/\mathrm{time}\;\left(h\right)$$

The apparent permeability coefficient (P_app_) of HA was calculated from the transepithelial flux of HA.

### 2.5 Transepithelial Electrical Resistance (TEER) Measurements

TEER values were measured to evaluate the integrity of the TR146 cell layers. TEER values were determined based on electrical resistance (*R,* measured in Ω), using an epithelial voltohmmeter (EVOM 2, World Precision Instruments, Sarasota, FL, USA) fitted with an STX-2 electrode, following the manufacturer’s protocol. Consistent with established protocols for oral epithelial models, a minimum TEER threshold of 80 Ω·cm^2^ was applied to confirm cell layer integrity prior to treatment. This threshold falls within the range reported for differentiated TR146 cultures in previous studies,^21,22^ indicating adequate epithelial barrier formation before experimental exposure. Only inserts exhibiting stable TEER values above this threshold were included in subsequent experiments. The calculation of TEER value was performed according to eq 4:

4 $$\mathrm{TEER}=\left(R_{\left(\mathrm{insert}\;\mathrm{with}\;\mathrm{cells}\right)}-R_{\left(\mathrm{insert}\;\mathrm{without}\;\mathrm{cells}\right)}\right)\times A$$

where *R*_(insert with cells)_ is the resistance of the insert with cells, *R*_(insert without cells)_ is the resistance of the cell-free insert, and *A* is the surface area of the filter membrane (cm^2^).

### 2.6 FD4 Transport and Determination of Apparent Permeability Coefficient

Fully differentiated TR146 cell layers were used for the FD4 transport experiments to confirm the effect of the SHTP film. SHTP films were placed on the apical side of the transwell insert, followed by the addition of culture medium. The cell layers were then incubated for 24 h at 37 °C in a humidified incubator with 5% CO_2_. Following incubation, TR146 cell layers on transwell inserts were gently washed with HBSS to eliminate residual test compounds. To allow equilibration, HBSS was added to both the apical (0.5 mL) and basolateral (1.5 mL) sides for 10 min. The transport experiment was initiated by substituting the apical HBSS with a fresh HBSS containing 1 mg/mL FD4, which was maintained at 37 °C throughout the experiment. At 0.5, 1, 2, 4, 8, and 24 h, 100 μL samples were withdrawn from the basolateral side and immediately replenished with the same volume of fresh HBSS. The fluorescence intensity of FD4 in the collected samples was measured using a fluorescence spectrophotometer (Thermo Fisher Scientific) with excitation and emission wavelengths set at 485 and 535 nm, respectively. The apparent permeability coefficient (P_app_) was calculated using eq 5. The P_app_ was calculated using transport data obtained during the first 4 h of the experiment.

5 $$P_{app}\;(cm/s)=\frac{V}{AC_{0}}\frac{dC}{dt}$$

Here, *V* represents the volume of the basolateral side solution (1.5 mL), *A* denotes the surface area of the membrane (0.9 cm^2^), *C*_0_ is the initial concentration in the apical compartment, and *dC/dt* is the steady-state rate of change in basolateral concentration over time. To ensure dimensional consistency, both *C*_0_ and the basolateral concentration (*C*) were expressed in identical units (e.g., mmol/L or mg/mL), yielding P_app_ in the standard units of cm/s. The relative P_app_ of FD4 in TR146 cell layers after pretreatment with the films was calculated as a percentage (%) of the P_app_ observed in the control group (untreated cells).

### 2.7 Total RNA Extraction and Quantitative Reverse Transcription Polymerase Chain Reaction (RT-PCR)

To assess the effects of the SHTP film on the mRNA expression of TJ-related genes, TR146 cells were seeded at a density of 5 × 10^4^ cells/well in six-well plates. Total RNA was isolated using the RNeasy Mini Kit (Qiagen, Hilden, Germany), following the manufacturer’s instructions. RNA concentration was quantified using a NanoDrop spectrophotometer (Implen, Munich, Germany). 1 μg of total RNA was used for cDNA synthesis via reverse transcription using the AccuPower PCR Premix (Bioneer, Daejeon, Republic of Korea) and a high-performance thermal cycler (Biometra TAdvanced, Applied Biosystems, Thermo Fisher Scientific, USA). The synthesized cDNA was then amplified in a 20 μL reaction mixture containing 10 μL of Power SYBR Green PCR Master Mix (Applied Biosystems, Thermo Fisher Scientific, USA), 5.2 μL of sterile deionized water, and 2 μM of each primer specific for TJ-related genes (ZO-2, cingulin, marvelD3, claudin-1, claudin-3, and claudin-4), upstream signaling genes regulating TJ-related genes (AMPKα1, CaMKKβ, and LKB1), and internal control (β-actin). The primer sequences used in this experiments are as follows: ZO-2 forward primer, 5′-GCCAAAACCCAGAACAAAGA-3′ and reverse primer, 5′-ACTGCTCTCTCCCACCTCCT-3′; MarvelD3 forward primer, 5′-GAACCCCCTTCGGAGAGATA-3′ and reverse primer, 5′-CGGCAAGGACAAAGTAGGAG-3′; cingulin forward primer, 5′-GCTCCTGTTAGCTCGTGGTCC-3′ and reverse primer, 5′-GAAAAGGCTCAGTTGGCTTG-3′; Claudin-1 forward primer, 5′-GCACATACCTTCATGTGGCTCAG-3′ and reverse primer, 5′-TGGAACAGAGCACAAACATGTCA-3′; claudin-3 forward primer, 5′- CAACACCATTATCCGGGACT-3′ and reverse primer, 5′-CTTGGTGGCCGTGTACTTCT-3′; claudin-4 forward primer, 5′-CATCGGCAGCAACATTGTCAC-3′ and reverse primer, 5′-GCACCACGCAGTTCATCCATAG-3′; AMPKα1 forward primer, 5′-AAACCCACAGAAATCCAAACAC-3′ and reverse primer, 5′-CCTTCCATTCATAGTCCAACTG-3′; CaMKKβ forward primer, 5′-AGACCAGGCCCGTTTCTACT-3′ and reverse primer, 5′-GAAGATCTTGCGGGTCTCAG-3′; LKB1 forward primer, 5′-AACGGCCTGGACACCTTCT-3′ and reverse primer, 5′-CCCTTCCCGATGTTCTCAA-3′; β-actin forward primer, 5′-TGGCACCCAGCACAATGAA-3′ and reverse primer, 5′-CTAAGTCATAGTCCGCCTAGAAGCA-3′. The reaction conditions used on the QuantStudio 3 Real-Time PCR system (Applied Biosystems, Thermo Fisher Scientific, USA) were as follows: 95 °C for 10 min followed by 40 cycles of denaturation for 15 s at 95 °C, annealing for 60 s at 60 °C, and extension for 15 s at 72 °C. The relative quantification values of the target genes were determined using the 2^–ΔΔCt^ method. The expression values of the selected genes were normalized to that of β-actin, and the relative expression level in untreated cells was set to 1. All reactions were conducted in triplicates.

### 2.8 Protein Extraction and Western Blot Analysis

The expression levels of TJ-related proteins (ZO-2, cingulin, marvelD3, and claudin-4) and upstream signaling proteins regulating the TJ-related proteins (p-AMPKα1, CaMKKβ, and LKB1) in the TR146 cells treated with or without SHTP film for 24 h were detected using Western blotting. After HA film treatment, the cells were lysed using ice-cold radioimmunoprecipitation assay buffer (Sigma-Aldrich) containing protease inhibitors (PhosSTOP; Roche, Basel, Switzerland). Following centrifugation at 8,000 × *g* for 10 min, the supernatants were collected, and total protein concentrations were measured using the bicinchoninic acid (BCA) protein assay kit (Thermo Fisher Scientific, USA). Equal protein amounts (12 μg/lane) were loaded onto 4–12% Bis-Tris Bolt gels (Invitrogen, Thermo Fisher Scientific, USA) and separated by electrophoresis. Proteins were subsequently transferred to polyvinylidene difluoride (PVDF) membrane (Invitrogen, Thermo Fisher Scientific, USA), which were blocked with 5% skimmed milk in Tris-buffered saline containing 0.05% Tween 20 (TBS-T) for 1 h at room temperature. Membranes were incubated overnight at 4 °C with the following primary antibodies: ZO-2 (1:1000, Cell Signaling Technology, Danvers, MA, USA), cingulin (1:2000, Abcam, Cambridge, UK), marvelD3 (1:1000, Proteintech, Seoul, Republic of Korea), claudin-4 (1:1000, Invitrogen, Thermo Fisher Scientific), phospho (Thr^172^)-AMPKα1 (1:1000, Merck Millipore, Burlington, MA, USA), CaMKKβ (1:1000, Abcam, UK), LKB1 (1:1000, Abcam, UK) or β-actin (1:2000, Abcam, UK). After thorough washing with TBS-T, membranes were incubated for 1 h with horseradish peroxidase (HRP)-conjugated secondary antibodies: goat antirabbit IgG or horse antimouse IgG (1:3000; Cell Signaling Technology). Immunoreactive proteins were detected using the Pierce ECL Western blotting substrate (Thermo Fisher Scientific) and a western imaging system (CAS-400SM, Davinch-K, Seoul, Republic of Korea). In particular, for removing p-AMPKα1, the membrane was incubated in stripping buffer (Biosesang, Yongin, Republic of Korea) for 30 min, blocked, and then reprobed with a primary antibody against AMPKα1 (1:1000 dilution, Merck Millipore) overnight at 4 °C. The secondary antibody [(HRP-conjugated goat antirabbit immunoglobulin G (IgG)] was incubated for 1 h at room temperature. Proteins were quantified using the ImageJ software (National Institutes of Health, Bethesda, MD, USA). Cellular protein levels were normalized based on the ratio of their levels to that of β-actin and AMPKα1 (for p-AMPKα1). The normalized results are expressed as protein levels in SHTP film-treated cells relative to those in control cells without SHTP film pretreatment.

### 2.9 Oral Administration and Collection of Serum Samples

Six-week-old male Crl:CD­(SD) rats were purchased from ORIENT Bio Inc. (Seongnam, Republic of Korea). For adaptation, all rats were housed in plastic cages for 2 weeks under controlled housing conditions at the Advanced Radiation Technology Institute (ARTI), Korea Atomic Energy Research Institute (KAERI). All animal experimental procedures were approved by the Institutional Animal Care and Use Committee of KAERI (KAERI-IACUC-2024-006) and conducted in compliance with the Laboratory Animal Act of Korea and internationally recognized guidelines, including the NIH Guide for the Care and Use of Laboratory Animals. Only male rats were used in this study to minimize biological variability due to sex-based differences. SHTP films (50 mg/kg body weight) were applied to the oral mucosa of rats and allowed to dissolve completely. Blood samples were collected from the lateral tail veins of rats. Whole blood samples were centrifuged at 3000 × *g* for 20 min. Serum samples were stored at −20 °C until assays with the HA-ELISA kit were performed (Echelon Biosciences, Inc., USA).

### 2.10 Statistical Analyses

All data were analyzed using IBM SPSS Statistics software (version 21; IBM Corp., Armonk, NY, USA). Results are presented as mean ± standard error of the mean (SEM). To assess statistical differences among multiple groups, one-way ANOVA was conducted, followed by the Tukey–Kramer posthoc test when appropriate. For comparisons between two groups, the Student’s *t*-test was applied. A p-value less than 0.05 was considered indicative of statistical significance.

## 3 Results

### 3.1 Physical Properties of HA Films

To evaluate the physical properties of the SHTP and commercial HA films, tensile strength, elongation at break, mucoadhesive strength, and water absorption were measured. As shown in Table 1, the SHTP film exhibited higher tensile strength and greater elongation at break than commercial HA film, indicating superior mechanical robustness. In contrast, the commercial HA film showed higher mucoadhesive strength than SHTP film. Regarding water absorption, the SHTP film demonstrated markedly greater water uptake, reaching 815% within 60 s, whereas the commercial HA film absorbed only 162% over the same period (Figure 1).

**Table 1:** Physical Properties (Tensile Strength, Elongation at Break, and Mucoadhesive Strength) of HA Films^a^

	Tensile strength (MPa)	Elongation at break (%)	Mucoadhesive strength (MPa)
Commercial HA film	4.47 ± 0.33	6.20 ± 0.68	1.41 ± 0.01
SHTP film	89.43 ± 1.11	39.13 ± 1.62	0.87 ± 0.05

a Data are expressed as means ± SEM (*n* = 3). SHTP film, solid hyaluronic acid technology platform-based film.

**Figure 1:**
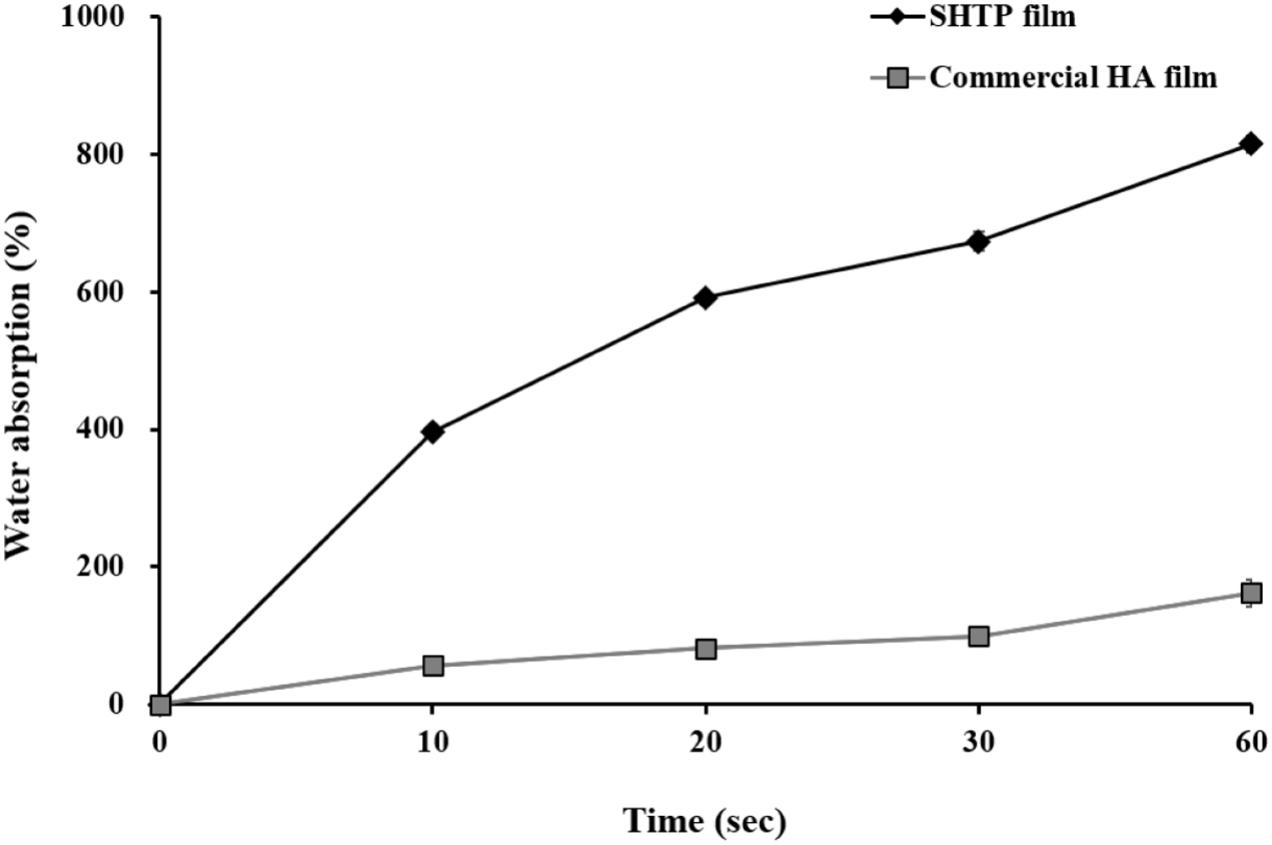
Water absorption of solid hyaluronic acid technology platform-based film (SHTP film) and commercial hyaluronic acid (HA) film. Water absorption profiles of the SHTP film and commercial HA film were measured at 10, 20, 30, and 60 s. Values are expressed as mean ± SEM (*n* = 3).

### 3.2 Permeation Profile of HA Films on TR146 Cell Layers

To compare the permeation characteristics of the SHTP and commercial HA films, HA permeation characteristics, including HA permeability, transepithelial flux, and apparent permeability coefficient (P_app_) were investigated (Figure 2 and Table 2). The permeability curves of the HA film samples across TR146 cell layers are shown in Figure 2A. The SHTP film, which had Manning level of 100% HA, showed higher permeability than the commercial HA film composed of HA and excipients, although commercial HA films contain considerable amount of HA per unit area (cm^2^) (Figure 2). As shown in Table 2, the transepithelial flux of HA and P_app_ of the SHTP film were also higher than those of the commercial HA film during 24 h. In particular, faster HA permeation was observed in the SHTP film-treated group than in the commercial HA film group during the first 1 h of the experiment (early phase) (Figure 2A and 2B).

**Figure 2:**
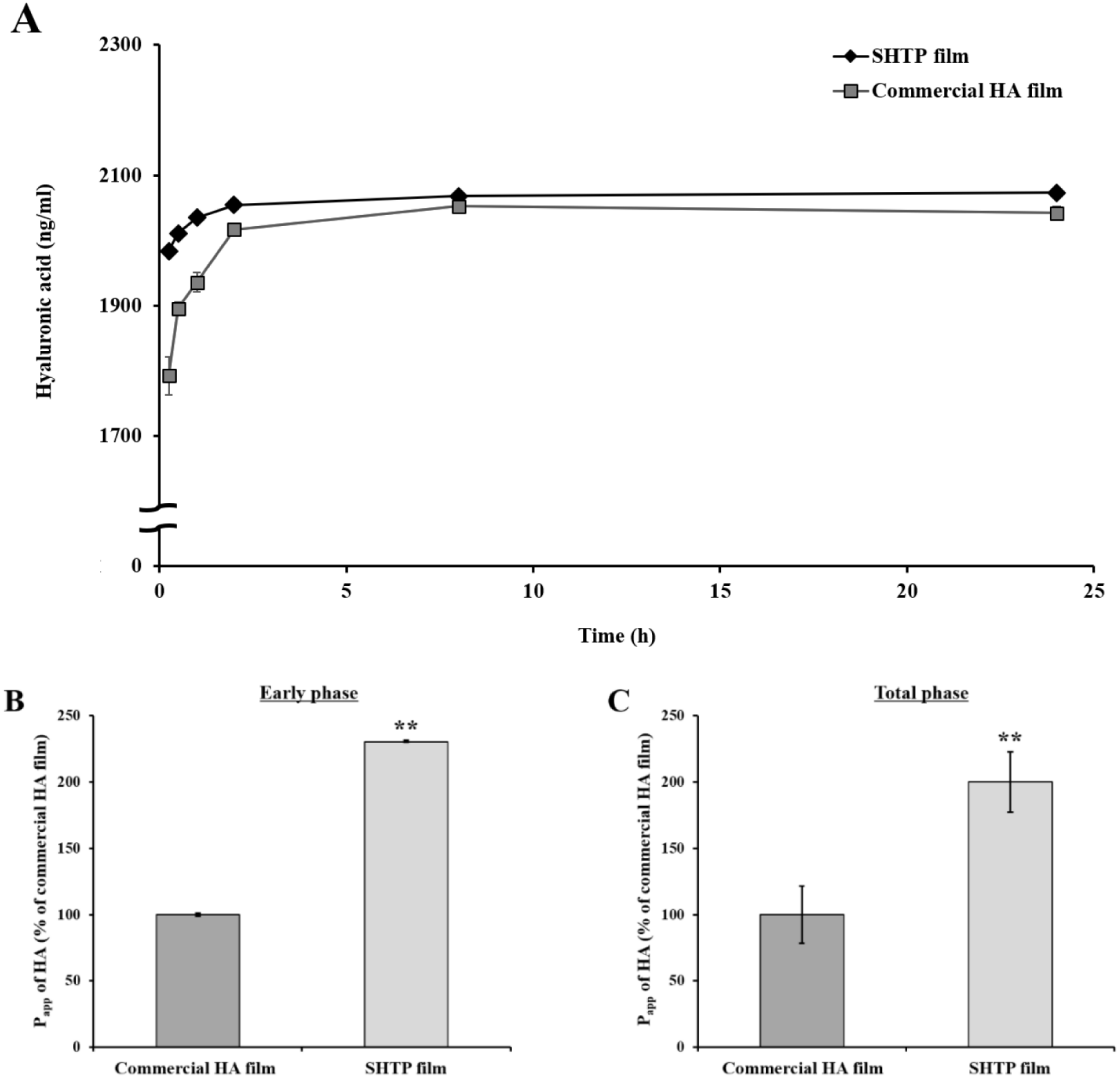
Permeation behavior and apparent permeability coefficient (P_app_) of HA from the SHTP film and commercial HA film across TR146 cell layers. TR146 cell layers were treated with HA films (SHTP film or commercial HA film). (A) Cumulative *in vitro* permeation profiles of HA across TR146 cell layers. (B) P_app_ of HA during the early phase (within 1 h) and (C) P_app_ of HA over the total phase (24 h). Values are expressed as mean ± SEM (*n* = 4). (**) *p* < 0.01 indicates significant differences from commercial HA film. Significant differences between the commercial HA film and SHTP film-treated groups were evaluated using the unpaired Student’s *t*-test.

**Table 2:** The Transepithelial Flux and Apparent Permeability Coefficients (P_app_) across TR146 Cell Layers of Commercial HA Film and the SHTP Film^a^

	Transepithelial flux (ng/cm^2^/h)		P_app_(cm/h·10^–4^)	
Time (h)	Commercial HA film	SHTP film	Commercial HA film	SHTP film
0.25	9604.22 ± 156.51	10627.36 ± 28.08	1.73 ± 0.028	4.28 ± 0.011
0.5	5077.06 ± 28.90	5387.08 ± 4.46	0.92 ± 0.005	2.17 ± 0.002
1	2592.74 ± 19.52	2725.30 ± 4.18	0.47 ± 0.004	1.10 ± 0.002
2	1350.77 ± 3.83	1375.79 ± 2.82	0.24 ± 0.001	0.55 ± 0.001
4	669.72 ± 9.34	680.50 ± 6.47	0.12 ± 0.002	0.27 ± 0.003
8	343.60 ± 0.86	346.20 ± 0.56	0.06 ± 0.000	0.14 ± 0.000
24	113.94 ± 0.57	115.70 ± 0.14	0.02 ± 0.000	0.05 ± 0.000

a Data are expressed as means ± SEM (*n* = 4). SHTP film, solid hyaluronic acid technology platform-based film.

### 3.3 Effect of SHTP Film on Oral Epithelial Barrier Integrity and Function

To demonstrate the effect of the SHTP film on oral barrier integrity and function, TR146 cell layers with or without the SHTP film were used. The ability of the SHTP film to modulate the integrity of the oral epithelial barrier was assessed using TEER values. To confirm that the SHTP film enhanced the function of the oral barrier, the effect of SHTP film on paracellular permeability was evaluated by measuring the apical-to-basolateral transport of FD4. The results (Figure 3) showed that after 24 h of exposure, the SHTP film reduced the permeability of FD4. Treatment with the SHTP film significantly increased the TEER value to 106 ± 0.65 Ω·cm^2^, corresponding to a 14% increase relative to the basal resistance (93 ± 0.96 Ω·cm^2^) observed in untreated control. These results suggested that the observed reduction in P_app_ following SHTP film treatment may be due to the SHTP film-induced enhancement of barrier function in the TR146 cell layers. To gain deeper insights, additional experiments were conducted using SHTP film-treated TR146 cells to analyze the gene expression changes linked to enhanced oral barrier integrity and function.

**Figure 3:**
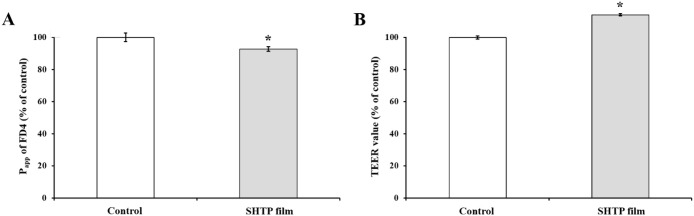
Effects of SHTP film on the P_app_values of FD4 and oral epithelial barrier integrity in TR146 cell layers. FD4 transport was investigated in TR146 cell layers treated with or without SHTP film for 24 h and compared with untreated cells (control). (A) P_app_ of FD4 across TR146 cell layers. (B) Transepithelial electrical resistance (TEER) as a measure of epithelial barrier integrity. Values are expressed as mean ± SEM (*n* = 4). (**) *p* < 0.01 indicate significant differences from untreated TR146 cells. Significant differences between the untreated (control) and SHTP film-treated groups were evaluated using the unpaired Student’s *t*-test.

### 3.4 Effect of SHTP Film on the Expression of TJ-Related Genes in TR146 Cells

TJ-related genes are involved in maintaining oral barrier function. Therefore, we detected the expression of TJ mRNA and protein using qRT-PCR and Western blot assays. The results are shown in Figures 4 and 5. SHTP film supplementation significantly increased the mRNA expression of ZO-2, cingulin, MarvelD3, claudin-1, claudin-3, and claudin-4, which are well-recognized TJ-related genes, suggesting that the SHTP film induces oral barrier enhancement in TR146 cells. At the protein level, the SHTP film treatment significantly increased the protein levels of ZO-2, cingulin, MarvelD3, and claudin-4 (Figure 4). However, the protein abundance of claudin-1 and claudin-3 were not affected by the SHTP film (Figure 5). These results suggested that the SHTP film regulates the mRNA and protein expression of TJ-related genes compared to that in control cells.

**Figure 4:**
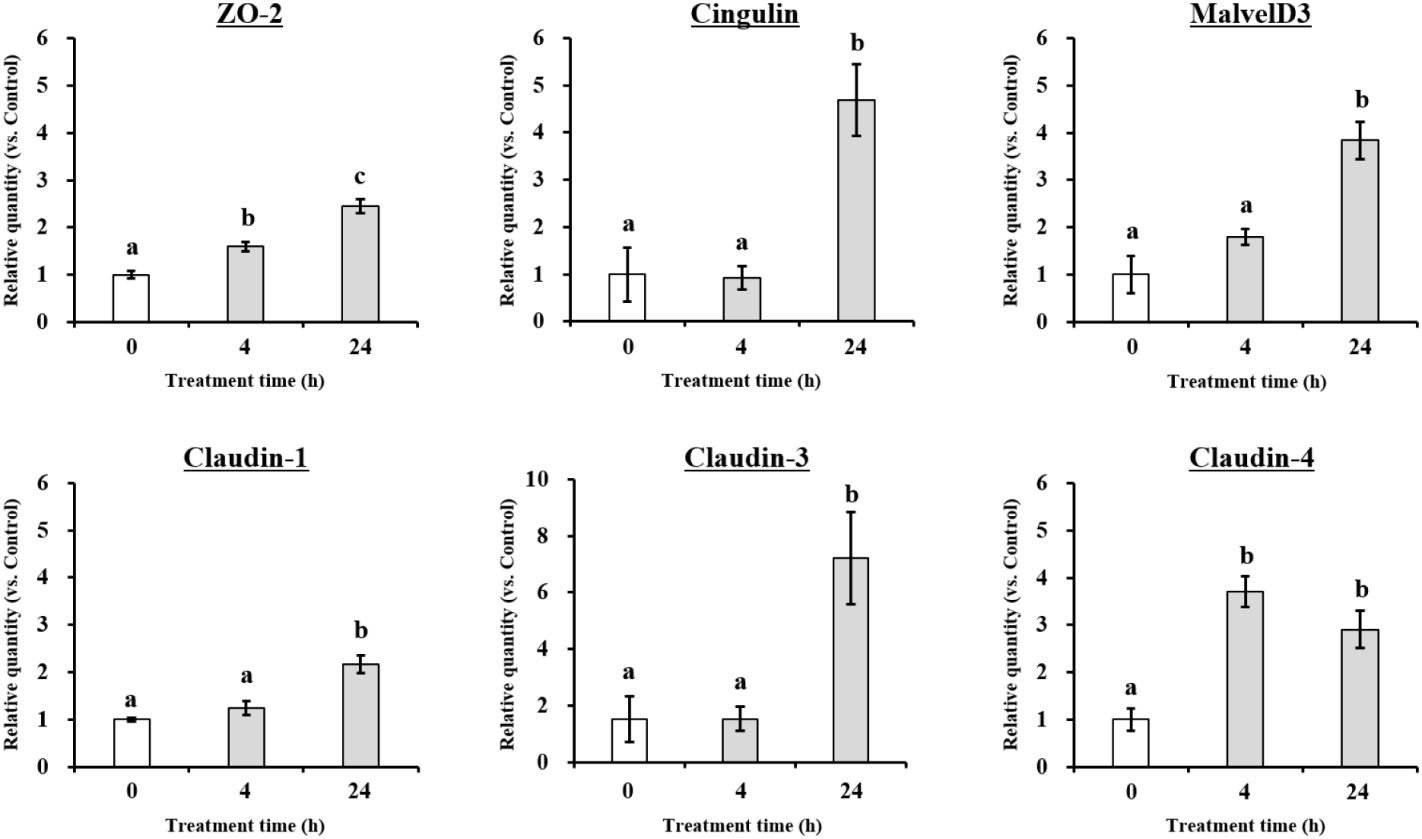
Effects of the SHTP film on the mRNA expression of tight junction (TJ)-related genes in TR146 cell layers. TR146 cells were treated with or without the SHTP film for 4 and 24 h. Total RNA was isolated and the mRNA levels of ZO-2, cingulin, MarvelD3, claudin-1, claudin-3, and claudin-4 were measured by qRT-PCR. β-actin was used as the internal control. Values are expressed as mean ± SEM (*n* = 3). Different letters indicate statistically significant differences (*p* < 0.05) between groups as determined using the Tukey–Kramer posthoc test.

**Figure 5:**
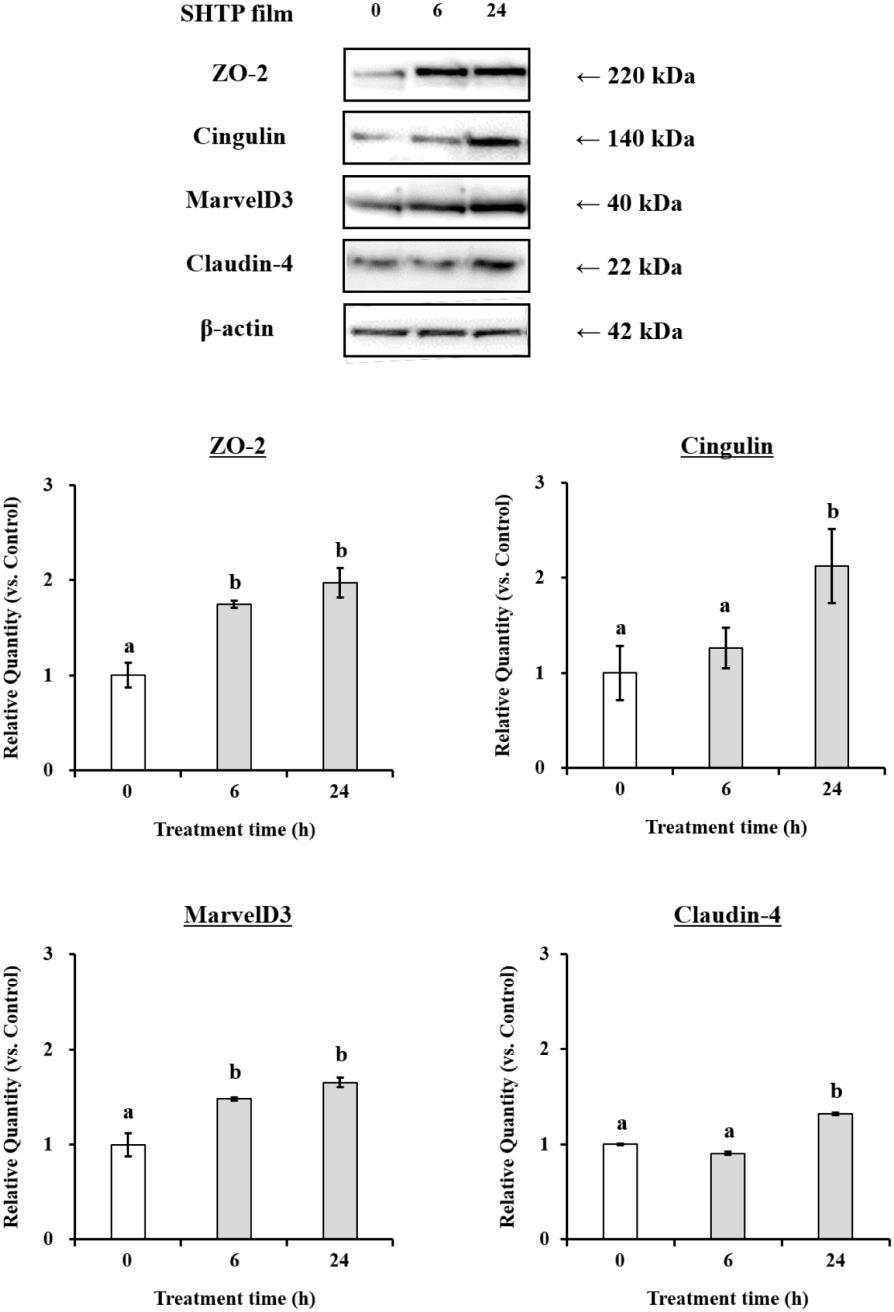
Effects of the SHTP film on TJ-related protein expression in TR146 cells. TR146 cells were treated with or without the SHTP film for 6 and 24 h. Levels of ZO-2, cingulin, MarvelD3, claudin-4 and β-actin (internal control) were quantified by densitometric analysis. Values are expressed as mean ± SEM (*n* = 3). Different letters indicate statistically significant differences (*p* < 0.05) between groups as determined using the Tukey–Kramer posthoc test.

### 3.5 Effect of the AMPK Pathway on SHTP Film-Induced Oral Barrier Enhancement

To investigate the upstream signaling via which the SHTP film induced enhancement of TJ-related gene expression, we focused on the AMPK signaling pathway. As shown in Figure 6, AMPKα1 mRNA was significantly upregulated in cells treated with the SHTP film. In the same manner, the phosphorylation level of AMPKα1 (p-AMPKα1) increased in SHTP film-treated TR146 cells compared with that in the untreated cells (control). These results showed that the SHTP film enhanced AMPK phosphorylation in TR146 cells, suggesting that the effect of the SHTP film on TJ-related protein expression may be mediated via the activation of the AMPK signaling pathway.

**Figure 6:**
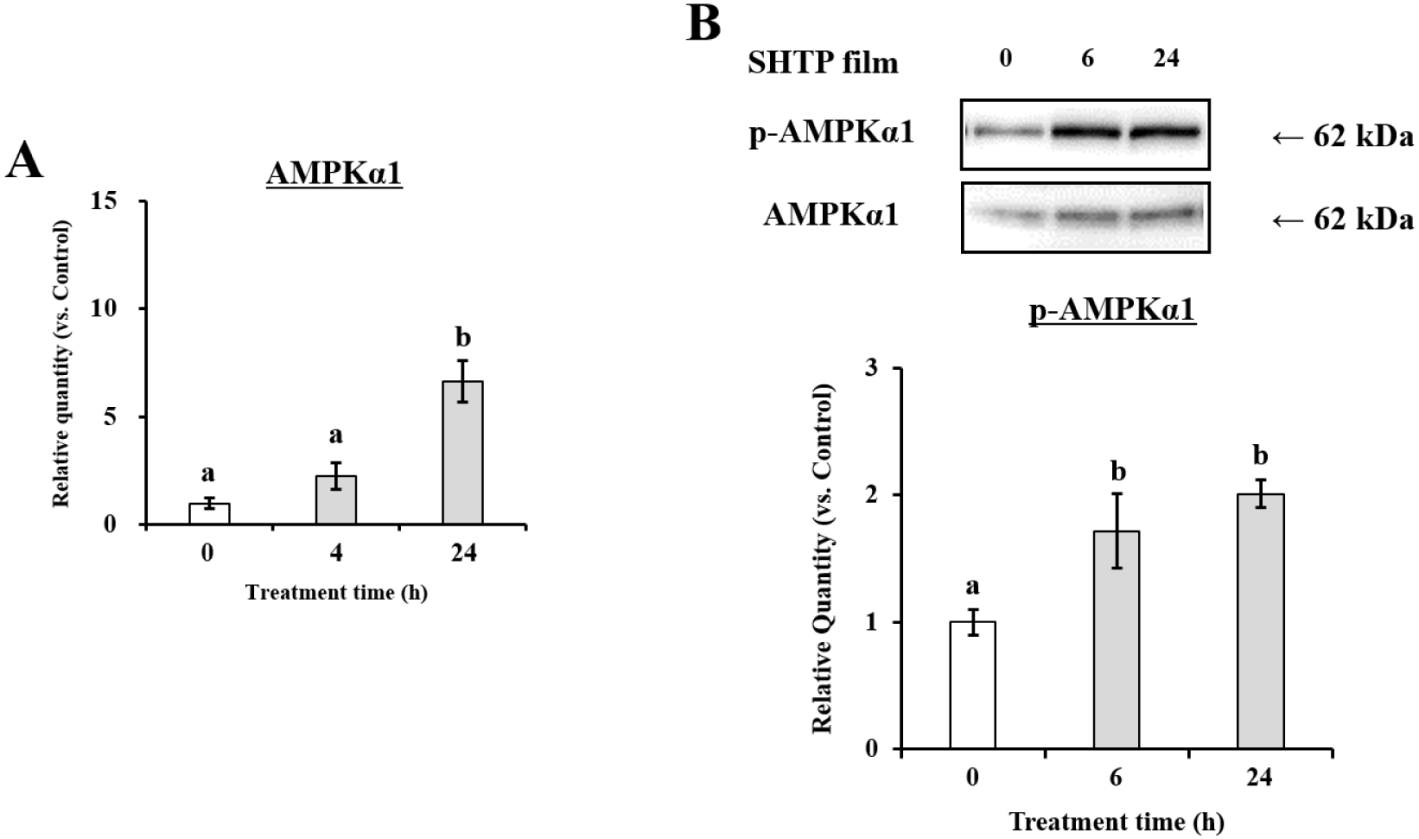
Effects of the SHTP film on phosphorylation of AMPK in TR146 cells. TR146 cells were treated with or without the SHTP film. (A) Total RNA was isolated and mRNA levels of AMPKα1 and β-actin were measured by qRT-PCR. (B) Bands specific for p-AMPKα1 and AMPKα1 were quantified using densitometric analysis. Values are expressed as mean ± SEM (*n* = 3). Different letters indicate statistically significant differences (*p* < 0.05) between groups as determined using the Tukey–Kramer posthoc test.

### 3.6 Involvement of the LKB1/CaMKKβ Pathway in SHTP Film-Induced Oral Barrier Enhancement

To further determine whether LKB1 and/or CaMKKβ contributes to SHTP film-promoted oral barrier function, we assessed the mRNA and protein levels of CaMKKβ and LKB1. As shown in Figure 7, LKB1 mRNA and protein levels increased after SHTP treatment. Consistently, SHTP film upregulated the CaMKKβ genes (Figure 8). These results indicated that SHTP film promoted oral barrier enhancement via the LKB1/CaMKKβ-AMPK pathway.

**Figure 7:**
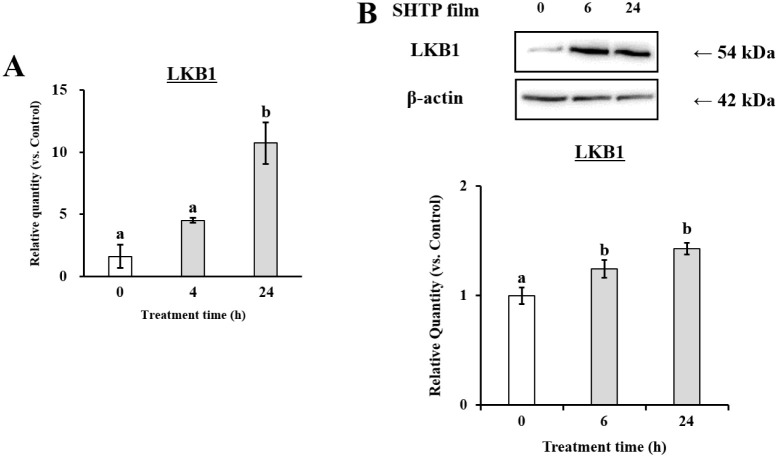
Effects of the SHTP film on LKB1 mRNA and protein expression in TR146 cells. TR146 cells were treated with or without the SHTP film. (A) Total RNA was isolated and the mRNA levels of LKB1 and β-actin were measured by qRT-PCR. (B) Bands specific for LKB1 and β-actin were quantified using densitometric analysis. Values are expressed as mean ± SEM (*n* = 3). Different letters indicate statistically significant differences (*p* < 0.05) between groups as determined using the Tukey–Kramer posthoc test.

**Figure 8:**
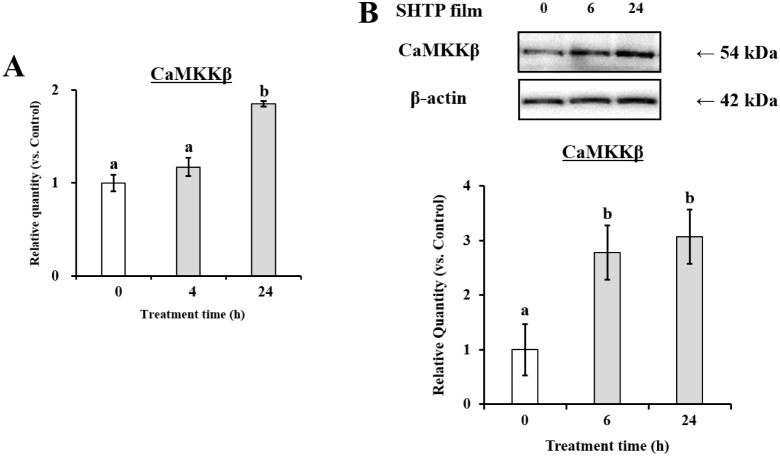
Effects of the SHTP film on CaMKKβ mRNA and protein expression in TR146 cells. TR146 cells were treated with or without the SHTP film. (A) Total RNA was isolated and mRNA levels of CaMKKβ and β-actin were measured using qRT-PCR. (B) Bands specific for CaMKKβ and β-actin were quantified using densitometric analysis. Values are expressed as mean ± SEM (*n* = 3). Different letters indicate statistically significant differences (*p* < 0.05) between groups as determined using the Tukey–Kramer posthoc test.

### 3.7 Oral Absorption of the SHTP Film in Rats

To confirm HA absorption through the oral route (oral-buccal absorption) *in vivo*, HA levels were measured in the sera of rats in which the SHTP film was applied to the oral mucosa. The HA concentration–time curves are shown in Figure 9. After application of the SHTP film, the absorbed HA appeared in the serum. In particular, the absorption increased significantly 20–30 min after SHTP film application. The HA amount increased approximately by 60% after 20 min (*p* < 0.01) and by 35% after 30 min (*p* < 0.05) of SHTP film treatment compared with the control. These findings confirmed the rapid absorption of the SHTP film via oral permeation, as evidenced by its detection in the serum.

**Figure 9:**
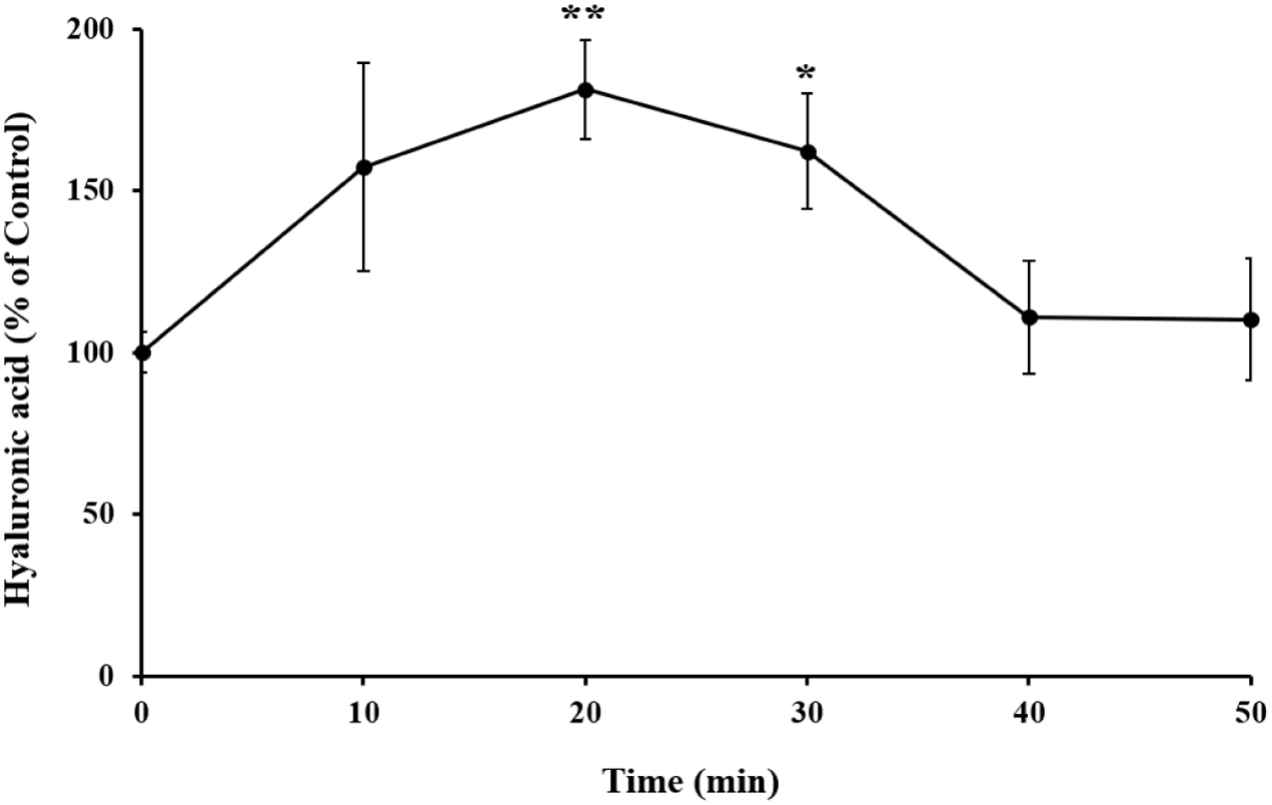
Permeation amount of HA from the SHTP film *in vivo*. SHTP films were applied to the buccal side of rat oral mucosa. Blood samples were collected from the lateral tail vein at 10, 20, 30, 40, and 50 min, and serum HA levels were measured using a hyaluronan enzyme-linked immunosorbent assay (ELISA). Values are expressed as mean ± SEM (*n* = 4). (*) *p* < 0.05 and (**) *p* < 0.01 indicate significant differences from untreated rats. Significant differences between the untreated (control) and SHTP film-treated groups were evaluated using the unpaired Student’s *t*-test.

## 4 Discussion

Oral films, characterized by their rapid dissolution in the oral cavity, were first introduced as breath-freshening products and have since evolved to meet broader needs by serving as convenient, portable, and patient-compliant functional ingredient delivery systems.^9^ Most edible oral films are formulated as composite polymeric matrices consisting of water-soluble polymers and various excipients to modulate mechanical strength, hydration behavior, and dissolution profiles.^10–14^ Recent studies have shown that excipients, while improving the physical and mechanical properties of the film, may inadvertently hinder the release or epithelial transport of bioactive compounds by forming dense polymeric networks that limit hydration or alter mucosal interactions.^15–17^

In line with these formulation-dependent behaviors, our physical evaluation revealed clear differences between the SHTP and commercial HA films. The SHTP film exhibited higher tensile strength and greater elongation at break, indicating superior mechanical robustness compared with the commercial HA film (Table 1). Conversely, the commercial HA film showed higher mucoadhesive strength, a finding consistent with previous reports that excipients such as pullulan or glycerin can increase mucosal adhesion by enhancing polymer chain entanglement with mucin.^15,23^ Notably, the SHTP film absorbed water much faster, reaching over five times the level of the commercial HA film at 60 s (815% vs 162%) (Figure 1). Such rapid hydration is known to promote early matrix swelling and disintegration, facilitating the initial release of hydrophilic polymers from oral films.^24^ These combined physical characteristics likely contribute to the enhanced early stage permeation behavior observed for the SHTP film in buccal epithelial layers.

HA, a glycosaminoglycan composed of repeating units of glucuronic acid and N-acetylglucosamine, has been frequently used in edible film formulations because of its excellent biocompatibility and mucoadhesive properties. In addition to its favorable physicochemical characteristics, HA is also known for its physiologically beneficial properties such as wound-healing and tissue hydration.^1–3,10^ Despite these advantages, the oral permeation behavior and epithelial barrier-modulating potential of excipient-free HA films remain insufficiently characterized, particularly in the context of oral mucosal delivery.

Accordingly, in the present study, the excipient-free SHTP film composed entirely of HA was evaluated for oral buccal permeation and barrier modulation and compared with a commercial HA film containing excipients (pullulan, glycerin, and modified starch). The effect of the SHTP film on oral epithelial barrier function and the underlying molecular mechanisms were investigated using human buccal TR146 cell layers, and the translational relevance of these findings was further examined through *in vivo* buccal absorption studies in rats.

TR146 cell layers are widely used as an *in vitro* model for investigating oral epithelial permeability and barrier function due to their ability to form stratified, nonkeratinized epithelial layers resembling human buccal mucosa.^22,25–27^ This model therefore provides a relevant platform for assessing both transepithelial permeation and barrier-regulatory responses induced by oral film formulations.

Compared with commercial HA films containing excipients, the excipient-free SHTP films exhibited significantly higher transepithelial HA flux and apparent permeability coefficients (P_app_) in TR146 cell layers (Figure 2 and Table 2). Notably, the P_app_ values for the SHTP film were approximately 2.5-fold higher than those of the commercial film during the first 15 min of the permeation assay (Table 2). This rapid permeation behavior is likely attributable to formulation-dependent differences in hydration and matrix structure. Previous studies have shown that excipients such as pullulan, glycerin, and modified starch can form viscous or gel-like networks that modulate dissolution kinetics and retard early diffusion of hydrophilic compounds.^15–17^ Although molecular weight can influence the physicochemical properties of HA films, the ∼19 kDa difference between the SHTP and commercial HA films remains within the low-molecular-weight range. Previous studies have further shown that an HA sample with a substantially higher molecular weight (∼88 kDa) can still exhibit greater transepithelial flux across TR146 cell layers than the 29 kDa commercial HA film from 0.5 h onward.^5^ This observation suggests that molecular weight differences alone may not fully account for the observed differences in hydration and permeation. Accordingly, the absence of such excipients in the SHTP film likely enables faster hydration-driven HA release and early epithelial permeation. In addition, differential effects on epithelial barrier function further indicate that matrix-dependent factors contribute to these differences, alongside potential molecular weight-related effects. While the SHTP film significantly reduced paracellular permeability, the commercial HA film did not exhibit a comparable barrier-enhancing effect and instead showed a trend toward increased FD4 permeability under the present experimental conditions. Notably, this increase in FD4 permeability was not accompanied by a decrease in TEER, suggesting that the epithelial barrier was not globally compromised (Figure S2). Rather, this pattern may reflect a selective modulation of paracellular transport, rather than overt barrier disruption. These findings suggest that molecular weight alone cannot fully account for the observed differences, and that formulation-specific matrix properties, including differences in hydration and dissolution behavior, also contribute to epithelial interactions and transport behavior.

While the present model reflects a dissolution-driven exposure process in which film-derived HA interacts with epithelial cells following in situ hydration, the relative contributions of film-derived HA and fully dissolved HA are not fully distinguished in this system. In addition, the relative contributions of molecular weight, dissolution behavior, and formulation characteristics cannot be fully separated under the current experimental conditions. Nevertheless, the comparative differences observed between the film systems under identical experimental conditions support a contribution of formulation-dependent behavior. Furthermore, although the permeation profile suggests a time-dependent exposure pattern, the present study does not constitute a formal dose–response analysis. This aspect should therefore be considered when interpreting the results.

To evaluate the effects of the SHTP film on epithelial barrier function following this initial permeation phase, paracellular permeability was assessed using fluorescein isothiocyanate-dextran (FD4), a commonly used macromolecular probe for TJ-mediated transport, together with TEER measurements. As shown in Figure 3, TR146 cell layers treated with the SHTP film exhibited significantly reduced FD4 transport and increased TEER values, indicating reinforcement of epithelial barrier integrity. These findings suggest that the SHTP film elicits a favorable epithelial response following initial HA permeation, thereby contributing to enhanced barrier function rather than causing barrier disruption during the subsequent epithelial response phase. Importantly, the enhanced early permeation of HA and the subsequent reinforcement of epithelial barrier integrity are not contradictory phenomena. Rather, these processes appear to occur sequentially, in which rapid initial HA permeation is followed by adaptive epithelial responses that strengthen TJ organization and barrier stability.

To further elucidate the molecular basis of this barrier-supportive response, the expression of TJ-related genes was examined. TJs are essential regulators of epithelial barrier integrity, controlling paracellular permeability and maintaining epithelial homeostasis.^28^ Mechanistically, the SHTP film upregulated the expression of key TJ components, including ZO-2, cingulin, MarvelD3, and claudin-4, at both the mRNA and protein levels (Figures 4 and 5), consistent with TJ tightening and improved intercellular cohesion. In contrast, claudin-1 and 3 showed increased mRNA expression, without corresponding changes at the protein levels, suggesting post-transcriptional regulation or delayed protein turnover.^29,30^ Such differential regulation indicates that HA-induced barrier modulation involves active cellular signaling rather than purely passive physical effects.

Given the established role of AMP-activated protein kinase (AMPK) in regulating epithelial barrier assembly and TJ organization,^31,32^ we further investigated AMPK signaling as a potential upstream mechanism. The SHTP film induced phosphorylation of AMPKα1 (Figure 6), accompanied by upregulation of its upstream kinases LKB1 and CaMKKβ (Figures 7 and 8). AMPK activation occurs via phosphorylation at the Thr-172 residue of its α subunit, mediated by LKB1 or CaMKKβ in response to elevated AMP/ATP ratios or increased intracellular calcium levels, respectively.^33,34^ Taken together, these findings are consistent with the involvement of both metabolic (LKB1-AMPK pathway) and calcium-sensitive (CaMKKβ-AMPK pathway) signaling pathways in SHTP film-induced TJ regulation. However, the LKB1/CaMKKβ-AMPK axis observed in this study should be considered an associated signaling response under the present experimental conditions, and causal involvement will require further validation using pharmacological inhibition or genetic knockdown approaches. Previous studies have linked HA to signaling pathways such as CD44-mediated ERK1/2 activation or TLR-related MyD88- NF-κB signaling,^35–37^ primarily in the context of wound healing or anti-inflammatory responses. HA is also known to interact with cell surface receptors such as CD44 and Toll-like receptors in epithelial tissues. However, receptor-mediated signaling was not directly examined in the present study, and therefore such mechanisms remain speculative. Within this context, the present findings support an association between excipient-free HA (SHTP) film exposure and AMPK-related regulation of epithelial barrier integrity and function. Nevertheless, given the complexity of epithelial barrier regulation, this pathway is likely to operate as part of a broader signaling network rather than as a standalone mechanism. Further studies are warranted to delineate the integrated molecular landscape and identify additional pathways that may complement the AMPK-associated mechanism in this delivery system.

It should be noted that rat oral mucosa is partially keratinized and therefore differs histologically from the nonkeratinized human buccal epithelium.^21,38^ Accordingly, the *in vivo* experiment in the present study was intended primarily as a proof of concept to confirm that the SHTP platform can facilitate systemic HA delivery under physiological conditions. In line with the *in vitro* permeation findings, *in vivo* studies demonstrated that HA delivered via the SHTP film was detectable in systemic circulation as early as 20 min following buccal administration (Figure 9). This absorption profile contrasts with previous reports of delayed systemic uptake following conventional oral administration of HA, which typically occurs after 1 h or later and is often limited to very low-molecular-weight HA species (such as those with molecular weight of 2 kDa).^39,40^ For example, Oe et al. reported that the maximal level of ^14^C-labeled HA (920 kDa) was reached 8 h after a single oral administration of 25 mg/kg ^14^C-labeled HA in SD rats.^39^ According to Sato et al., the plasma concentration increased from 1 to 8 h in Wistar rats after oral administration of 200 mg/kg of 2 kDa HA.^40^ These observations suggest that the SHTP platform may facilitate rapid buccal absorption while maintaining epithelial barrier integrity.

Collectively, this study highlights the dual functional characteristics of the excipient-free SHTP film, enabling rapid transepithelial HA permeation followed by reinforcement of oral epithelial barrier function through TJ regulation. The present findings provide evidence supporting an association between excipient-free HA (SHTP) film exposure and LKB1/CaMKKβ-AMPK-associated modulation of epithelial barrier integrity and function. Further comparative *in vivo* studies are warranted to delineate formulation-specific contributions and to validate the translational potential of the SHTP platform as an oral epithelial barrier-oriented delivery and therapeutic system. Such investigations may further clarify the functional advantages of excipient-free HA films and support their potential application in oral biomedical and epithelial barrier-oriented delivery strategies.

## 5 Conclusions

This study demonstrates that an excipient-free solid hyaluronic acid technology platform (SHTP) film, composed entirely of HA, possesses distinctive physical properties, including higher tensile strength, greater elongation at break, and markedly faster water absorption (>5-fold relative to the commercial film at 60 s). These characteristics reflect rapid hydration behavior coupled with adequate structural integrity, providing a physicochemical basis for its oral performance. Building on these material attributes, the SHTP film demonstrated superior oral permeation compared with a commercial HA film containing conventional excipients. In TR146 cell layers, the excipient-free SHTP film first facilitated efficient transepithelial HA transport, as reflected by increased HA flux and apparent permeability. Following this permeation phase, the SHTP film elicited a barrier-supportive epithelial response, characterized by elevated transepithelial electrical resistance (TEER) values and reduced paracellular FD4 flux. These barrier-supportive effects were associated with upregulation of TJ-related genes and activation of the LKB1/CaMKKβ-AMPK signaling pathway.

Consistent with the *in vitro* findings, the *in vivo* results indicated that HA delivered via the SHTP film undergoes rapid systemic absorption following buccal administration. Taken together, these results highlight the dual functional potential of the SHTP film, supporting both efficient buccal transepithelial delivery and reinforcement of oral epithelial barrier integrity. To better delineate formulation-specific contributions and underlying mechanisms, further comparative *in vivo* studies between excipient-free and conventional excipient-based HA films are warranted. Such investigations will help clarify the functional advantages of the SHTP platform and support its potential application as an oral delivery and therapeutic platform.

## Supplementary Material



## Data Availability

All data supporting the findings of this study are available within the article and its Supporting Information.

## Notes

Figure S1: molecular weight characterization of hyaluronic acid (HA) used for the preparation of the SHTP film by gel permeation chromatography (GPC); Figure S2: differential effects of SHTP film and commercial HA film on the apparent permeability (P_app_) of FD4 and oral epithelial barrier integrity in TR146 cell layers (PDF)
