# Supplementary material for: Physicochemical Attributes of Solid Hyaluronic Acid Technology Platform-Based Film Drive Rapid Oral Permeation and Barrier Enhancement via LKB1/CaMKKβ-AMPK Signaling
Source: ACS Omega. 2026 May 18;11(21):31027–39. doi: 10.1021/acsomega.6c00470 (PMC13234882; doi:10.1021/acsomega.6c00470)
Supplement: Supplementary file 1 [file ao6c00470_si_001.pdf]

Supporting Information

Physicochemical Attributes of Solid Hyaluronic Acid Technology Platform-Based Film Drive Rapid Oral Permeation and Barrier Enhancement via LKB1/CaMKKβ-AMPK Signaling

Ha-Young Park[a,*], Soo-Bin Shin [a,b] and Dong-Keon Kweon[c]

[a]Advanced Radiation Technology Institute, Korea Atomic Energy Research Institute, Jeongeup 56212, Republic of Korea

[b]Department of Integrative Food, Bioscience and Biotechnology, Chonnam National University, Gwangju 61186, Republic of Korea

[c]Jinwoo Bio Co., Ltd., Giheung-gu, Yongin 17111, Republic of Korea

*Corresponding authors

E-mail addresses: hypark@kaeri.re.kr (H.-Y. Park)

Figure S1.

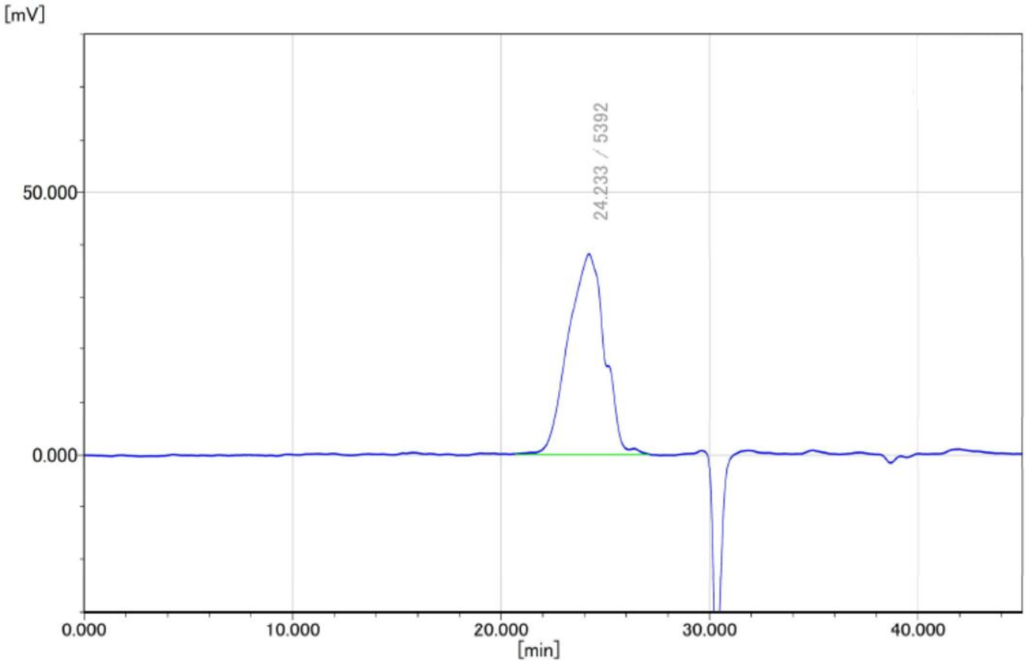

Figure S1. Molecular weight characterization of hyaluronic acid (HA) used for the preparation of the SHTP film by gel permeation chromatography (GPC). The molecular weight distribution of HA used as the starting material for the SHTP film was analyzed by GPC using a refractive index detector (HLC-8420 GPC system, Tosoh, Japan). Separation was performed with a TSKgel column set consisting of a PWxl guard column and GMPWxl and G2500PWxl analytical columns using 0.1 M $NaNO_3$ as the mobile phase at a flow rate of 1.0 mL/min and a column temperature of 40 ℃. The chromatogram showed a single dominant peak with a peak molecular weight (M_p) of approximately 5.4 kDa (5392 Da), with no evidence of distinct secondary populations.

Figure S2.

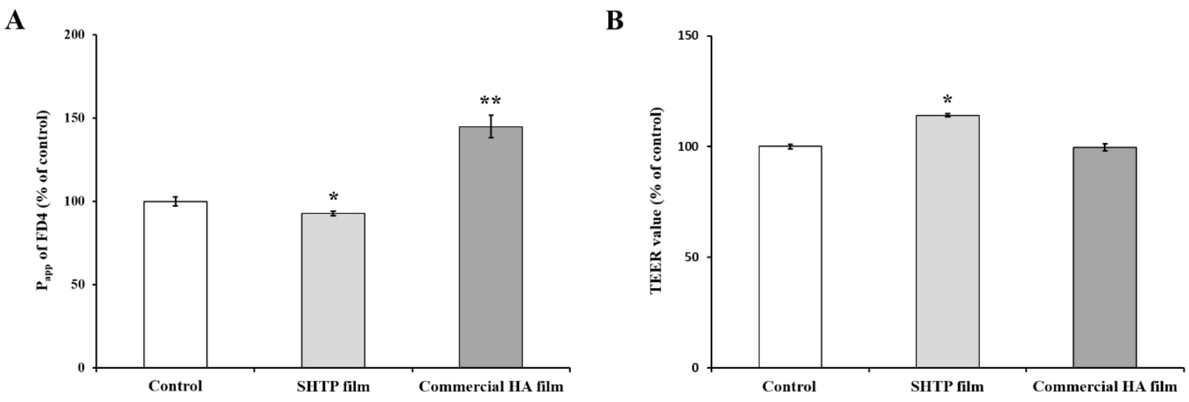

Figure S2. Differential effects of SHTP film and commercial HA film on the apparent permeability (P_{app}) of FD4 and oral epithelial barrier integrity in TR146 cell layers. FD4 transport was evaluated in TR146 cell layers after 24 h treatment with SHTP film or commercial HA film, compared with untreated control cells. (A) P_{app} of FD4 across TR146 cell layers. (B) Transepithelial electrical resistance (TEER) as an indicator of epithelial barrier integrity. Data are expressed as mean ± SEM (n = 4). Statistical significance was determined using un unpaired Student's *t*-test, where (*) $p < 0.05$ and (**) $p < 0.01$ indicate significant differences compared with the control.
